# Behavioral Determinants of Lifestyle Modifications Following Metabolic Bariatric Surgery in a Culturally Specific Population: A Qualitative Study

**DOI:** 10.1007/s11695-026-08941-6

**Published:** 2026-09-15

**Authors:** Haya Subhi, Rana Hijazi, Mahmoud Abu Gazala, Husam Mahajna, Ram Elazary, Tair Ben-Porat

**Affiliations:** 1 School of Public Health, The Faculty of Social Welfare and Health Sciences, University of Haifa, Haifa, Israel; 2 Department of Surgery, Hadassah Medical Center, Jerusalem, Israel; 3 Levinsky-Wingate Academic College, Netanya, Israel; 4 Faculty of Medicine, Hebrew University of Jerusalem, Jerusalem, Israel

**Keywords:** Obesity, Metabolic bariatric surgery, Behavior change, Qualitative research, COM-B model, Theoretical Domains Framework

## Abstract

**Introduction:**

Sustained healthy behaviors are critical for long-term success after metabolic bariatric surgery (MBS); however, patients from culturally distinct communities may face unique sociocultural and structural barriers to postoperative behavior change. We explored behavioral determinants of lifestyle modifications among women from the Arab society in Israel post-MBS.

**Methods:**

Semi-structured, in-depth interviews were conducted with 20 women who underwent MBS at a public university-affiliated medical center. Interviews explored participants’ experiences of postoperative lifestyle adjustments, including dietary and physical activity patterns. Data were analyzed using inductive thematic analysis followed by deductive mapping to the COM-B model and the Theoretical Domains Framework (TDF).

**Results:**

Seventeen themes were identified across all COM-B components and 12 TDF domains. Key facilitators included knowledge of dietary recommendations; improved physical functioning and stamina; and social support as an enabler of behavior change. Motivation was reinforced by health-related consequences and concerns of relapse; and visible progress and external feedback. Major barriers included limited postoperative follow-up and healthcare access; time, resource, and infrastructure constraints to physical activity. Additional challenges included variability in physical activity guidance; social norms, pressure, and expectations; and postoperative emotional distress and adjustment. Participants also described self-directed learning and information seeking, including the use of AI and digital tools.

**Conclusion:**

Postoperative lifestyle adherence following MBS is shaped by interacting individual, social, and environmental factors. Findings highlight the need for culturally tailored, theory-informed interventions integrating structured guidance, continuous follow-up, social support, and potentially digitally supported strategies to address real-world and culturally embedded barriers and support long-term outcomes following MBS.

**Supplementary Information:**

The online version contains supplementary material available at https://doi.org/10.1007/s11695-026-08941-6.

## Key points

• Adoption of healthy lifestyle modifications following MBS is shaped by social, cultural, and healthcare system contexts.

• Gaps in follow-up and time, resource, and infrastructure constraints are key-perceived barriers to sustained healthy lifestyle changes among Arab women.

• Social support from family, peers, and healthcare providers facilitates healthy lifestyle behavior change.

• Visible health improvements and concerns of relapse reinforce ongoing adherence.

**Supplementary Information:**

The online version contains supplementary material available at https://doi.org/10.1007/s11695-026-08941-6.

## Introduction

Obesity is a chronic, relapsing disease associated with substantial morbidity and an increased risk of premature mortality [1]. In Israel, approximately one quarter of adults live with obesity, with a higher prevalence among women from the Arab society, who experience elevated rates of obesity-related comorbidities including type 2 diabetes [2–4]. Metabolic bariatric surgery (MBS) is the most effective intervention for severe obesity, producing significant and durable weight loss and metabolic improvement [5]. However, long-term surgical success relies heavily on sustained adherence to postoperative lifestyle recommendations, particularly the adoption of healthy dietary and physical activity patterns [6, 7]. Weight recurrence and suboptimal metabolic outcomes remain clinically significant challenges, with evidence highlighting the important role of behavioral determinants in shaping long-term health outcomes [6, 8].

While adjunct behavioral interventions can enhance postoperative outcomes, many existing lack structured theoretical foundation and cultural adaptation [9–12]. This gap is particularly relevant among high-risk populations, such as women from the Arab society in Israel who may face unique sociocultural and structural factors influencing their ability to adopt and sustain recommended lifestyle behaviors following MBS [13, 14]. For example, Arab women in Israel report comparatively low levels of physical activity and dietary patterns characterized by higher carbohydrate and sugar intake, alongside substantially higher rates of food insecurity [3, 13]. Furthermore, previous research has shown that Arab women undergoing MBS had significantly higher rates of micronutrient deficiencies, particularly iron and vitamin D deficiencies, than Jewish women [15]. These disparities have been attributed to differences in dietary patterns, health literacy, socioeconomic conditions, and cultural practices, including reduced sun exposure associated with traditional clothing [15]. Collectively, the observed behavioral and nutritional disparities contribute to the disproportionately high burden of obesity and related morbidity among Arab women and emphasize the need for targeted, culturally appropriate interventions.

Frameworks for complex intervention development, such as the Medical Research Council (MRC) guidelines and the Obesity-Related Behavioural Intervention Trials (ORBIT) model, emphasize the importance of early-stage research to identify behavioral determinants to inform intervention design [16, 17]. The COM-B model (Capability, Opportunity, Motivation - Behavior) posits that behavior is influenced by the interaction of three key components each divided into two constructs: capability, comprising physical and psychological capability, refers to an individual’s ability to perform a behavior; opportunity, comprising physical and social opportunity, refers to external factors that enable or constrain behavior; and motivation, comprising reflective and automatic motivation, refers to cognitive and emotional processes that drive behavior [18, 19]. These components form an interacting system in which capability and opportunity can influence motivation, while motivation can lead to behavior that increases capability or opportunity [19]. The Theoretical Domains Framework (TDF) expands upon COM-B by providing 14 domains that offer more detailed insights of behavioral influences [20]. Further definitions of the COM-B constructs and the 14 TDF domains are provided in Appendix S1.

The present study represents the first phase of the STEP-UP research program (Supporting Theory-based Engagement to Promote Uptake of healthy lifestyle behaviors Post-MBS), a multi-phase initiative aimed at developing and piloting a theory-informed behavioral intervention as an adjunct to MBS. This qualitative phase sought to explore patient-reported facilitators, barriers, and behavioral support needs among women from the Arab society following MBS, with the aim of informing the development of a culturally responsive intervention integrated within multidisciplinary bariatric care.

## Methods

### Study Design

A qualitative study design was employed using semi-structured, in-depth interviews to explore behavioral determinants of lifestyle modification among women from the Arab society following MBS. Interviews were conducted between September 2025 and April 2026. The study followed the COnsolidated criteria for REporting Qualitative research checklist (Appendix S2) [21].

### Participants and Setting

Participants were women from the Arab society who underwent a primary MBS at Hadassah Ein-Kerem Medical Center, Jerusalem, Israel; a public, university-affiliated, urban hospital serving a diverse population. Eligibility criteria included: age 18–65 years and underwent MBS within the past 1–12 months. Exclusion criteria included: major postoperative complications affecting dietary intake or physical activity, prior MBS, or cognitive condition that could impair participation in an in-depth interview. Participants were recruited through medical records, clinician referrals, and follow-up visits using a purposive sampling strategy. Eligible individuals were contacted by phone by a member of the research team. Of 33 individuals contacted, 20 participated in the study, while 13 did not due to refusal (*n* = 3), discontinued contact (*n* = 3), or non-attendance at a scheduled interview (*n* = 7). Thematic saturation was reached after 14 interviews, and additional interviews were conducted to increase variation in participant characteristics and confirm that no new themes emerged.

### Data Collection

A semi-structured interview guide was developed based on the COM-B model and the TDF [20] (Appendix S3). Questions were open-ended, with probes used to explore participants’ postoperative lifestyle behaviors, perceived facilitators and barriers, and support needs. The guide was developed by a multidisciplinary team, including an Arabic-speaking PhD candidate who led the development of the interview guide (HS); a behavioral scientist to ensure theoretical alignment (TBP); a qualitative researcher to ensure methodological appropriateness (RH); and clinician-researchers from the MBS field (TPB, HS, HM, MAG, RE). Interviews were conducted one-on-one in Arabic via secure video conferencing (e.g., Zoom Pro) by the lead researcher (HS); a female PhD candidate trained in qualitative research, working within the bariatric clinic, with no prior relationship with participants. Interviews lasted approximately 30–60 min and were audio-recorded and transcribed verbatim. Demographic and surgery-related information was also collected. The lead researcher maintained reflexive field notes throughout data collection.

### Analysis

Analysis followed a two-stage process. First, an inductive analysis was conducted using a six-phase thematic analysis approach to identify emerging themes grounded in participants’ narratives [22]. Second, the inductively developed themes were mapped deductively onto COM-B constructs and the TDF domains to support theory-informed interpretation [19]. The theory-informed deductive phase was applied only after the inductive thematic structure had been developed, and was used to organize and interpret the empirically derived themes rather than guide the initial coding process. The analytic process comprised the following steps:

1. To gain familiarity with the data, the lead researcher (HS) transcribed the interviews verbatim and conducted the initial inductive coding manually. Related codes were grouped under preliminary thematic labels in a coding table. Regular meetings of the multidisciplinary research team (HS, RH, and TBP) were held to discuss emerging themes, refine the coding framework, and reach consensus on interpretation. Reflexivity was supported through discussion of researcher assumptions.
2. To enhance the trustworthiness of the analysis, an additional qualitative researcher (RH) independently coded 25% of the transcripts. HS and RH compared their coding and interpretations. Coding was broadly aligned, and any differences were discussed until consensus was reached. The coding framework and emerging themes were refined accordingly. This independent double-coding provided an additional perspective and strengthened the credibility of the analysis.
3. Following completion of the inductive analysis, the finalized themes were mapped onto the COM-B constructs and TDF domains using the published framework definitions. The proposed mappings were reviewed by the research team (HS, RH, and TBP). Where a theme could be mapped to more than one construct or domain, the supporting extracts and relevant framework definitions were reviewed, and the final mapping was agreed through consensus.
4. Following the initial theoretical mapping, all transcripts were reviewed again by HS to verify the alignment of the themes and supporting quotations with their assigned COM-B constructs and TDF domains and to ensure that no relevant findings had been overlooked. The final themes, supporting quotations, and theoretical mappings were reviewed, refined, and agreed upon by the research team.

### Ethical Considerations

The study was approved by the Institutional Review Board (IRB) of Hadassah Ein-Kerem Medical Center and the Faculty of Social Welfare and Health Sciences Ethics Committee for research with human subjects at the University of Haifa. All participants provided verbal informed consent prior to participation. This consent was documented by the researcher using a designated consent form in accordance with IRB requirements. Audio recordings and transcripts were stored securely on password-protected devices accessible only to the research team.

## Results

### Participant Characteristics

Twenty women living in East Jerusalem district participated in the study. Sociodemographic and clinical characteristics of participants are presented in Table 1.

**Table 1:** Sociodemographic and clinical characteristics of participants (*N* = 20)

Variable	*n* (%) or mean ± SD
Age (years)	35.5 ± 10.6
Marital status	
Married	15 (75%)
Single	4 (20%)
Widowed	1 (5%)
With children	14 (70%)
Education	
Middle school	4 (20%)
High school/professional certificate	13 (65%)
Academic degree	3 (15%)
Employment status	
Public employment	7 (35%)
No formal employment	13 (65%)
Type of surgery	
Sleeve gastrectomy	12 (60%)
Roux-en-Y gastric bypass	3 (15%)
One-anastomosis gastric bypass	5 (25%)
Postoperative time	
2–4 months	6 (30%)
5–8 months	10 (50%)
9–12 months	4 (20%)
BMI at interview (kg/m²)	32.1 ± 4.1
Preoperative BMI (kg/m²)	44.3 ± 4.7

### Themes

**Fig. 1:**
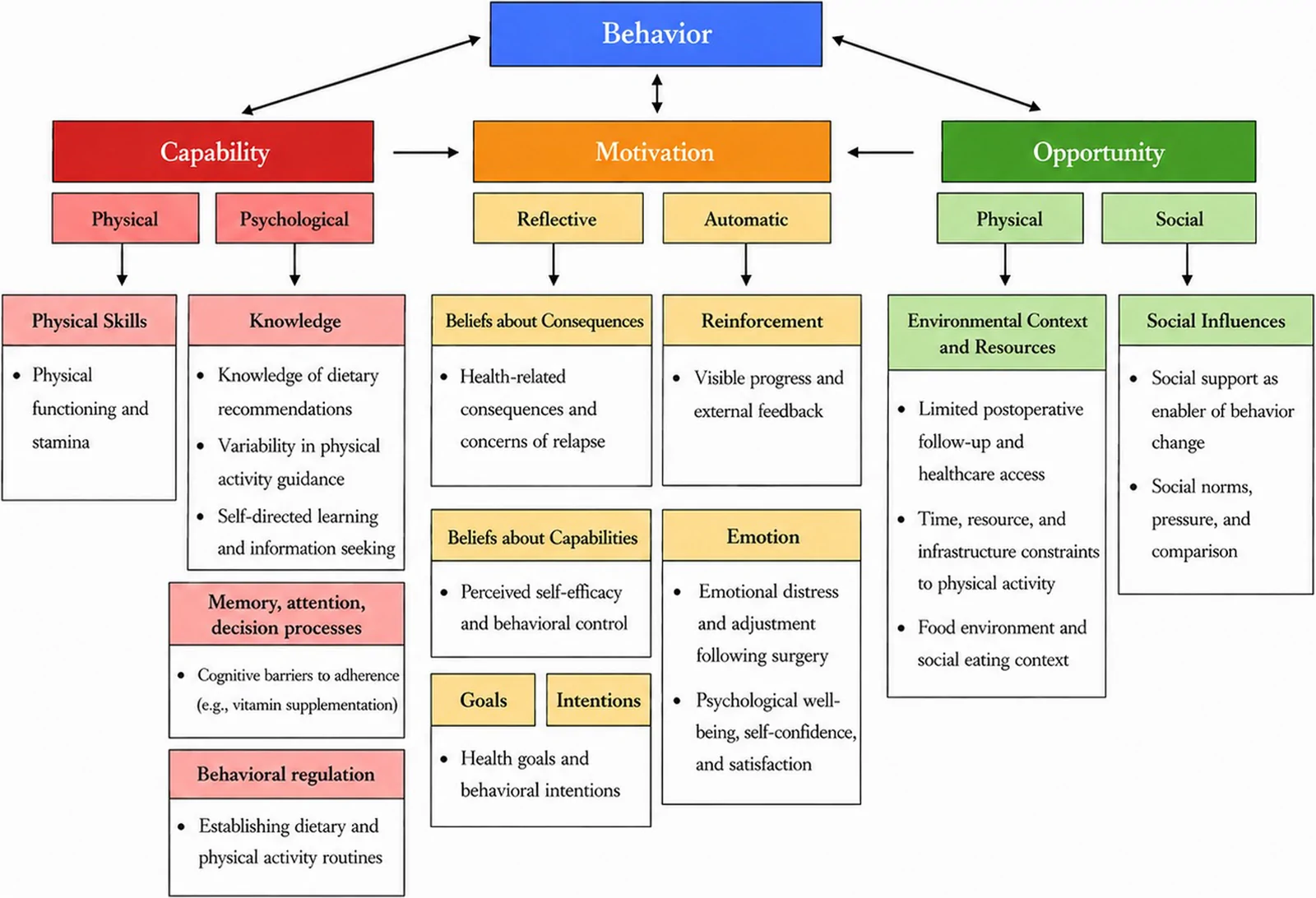
Behavioral determinants of lifestyle modification following metabolic bariatric surgery mapped to COM-B constructs and TDF domains. Abbreviations: COM-B, Capability–Opportunity–Motivation–Behavior; TDF, Theoretical Domains Framework. Note: COM-B represents an interacting system in which capability and opportunity influence motivation, and all three components interact to generate behavior

Inductive analysis identified themes reflecting postoperative experiences and perceived barriers and facilitators for lifestyle modifications post-MBS. These were mapped onto COM-B and TDF domains. Seventeen themes were identified across all six COM-B constructs and 12 of the 14 TDF domains, while optimism and social/professional role and identity were insufficiently represented in the data and therefore were not retained in the final findings **(**Fig. 1**)**. Illustrative participant quotations are presented in Table 2.

**Table 2:** Thematic findings mapped to COM-B constructs and TDF domains with illustrative participant quotations

Core theme	COM-B construct	TDF domain	Illustrative quotations^&^
Physical functioning and stamina	Physical capability	Physical skills	*“My movement increased so much. I walk*,* I laugh*,* I’m active*,* my family isn’t used to seeing me like this” P10*,* 32*
			*“After losing weight*,* everything feels lighter. Moving around the house is easier; before*,* I couldn’t last more than five minutes on the treadmill” P12*,* 50*
			*“My body was tired*,* I used to not be able to open a bottle*,* now I feel stronger and energized” P2*,* 28*
			*“My sleep changed completely*,* no snoring*,* no shortness of breath*,* uninterrupted sleep*,* I wake up rested” P15*,* 37*
			*“My sleep*,* my abdominal pain it all changed*,* I don’t feel the same pain anymore” P19*,* 23*
Knowledge of dietary recommendations	Psychological capability	Knowledge	*“My nutritionist told me to eat proteins in every meal like fish*,* chicken*,* also include vegetables*,* and take time to chew food” P10*,* 32*
			*“My nutritionist explained how to divide meals*,* what to start with*,* and not to mix eating and drinking*,* she gave me a program*,* and I am still following it properly” P16*,* 48*
			*“They instructed me not to mix water with food*,* to chew food as much as I can*,* eat small meals and focus on protein… all of that was clear” P17*,* 32*
Variability in physical activity guidance	Psychological capability	Knowledge	*“In the first three months they told me not to exercise*,* and then after three months I was allowed” P4*,* 50*
			*“My nutritionist told me mainly to walk*,* and not to do any abdominal exercises the first 3 months” P5*,* 53*
			*“They told me to walk*,* start light*,* then gradually stronger*,* and not to carry heavy weights at first” P9*,* 22*
			*“No one at all told me anything about physical activity…honestly*,* the information was lacking*,* I wish they would improve it” P19*,* 23*
Self-directed learning and information seeking	Psychological capability	Knowledge	*“I searched a lot at the beginning about what food suits me and what doesn’t” P11*,* 34*
			*“When I got stressed at the beginning*,* I turned to ChatGPT*,* I asked about constipation for example… I would even send photos of foods and ask if they were allowed” P16*,* 48*
			*“I ask ChatGPT a lot of questions… I also go into social groups online to see people’s opinions*,* but I rely more on ChatGPT because it explains more precisely and in detail without discouraging me” P17*,* 32*
Cognitive barriers to adherence (e.g., vitamin supplementation)	Psychological capability	Memory, attention, and decision processes	*“I’m very negligent with vitamins*,* it’s the only thing I forget” P9*,* 22*
			*“When the vitamins ran out*,* I started getting dizzy*,* I couldn’t get up*,* I had no energy*,* no strength. When I forget them even for a couple of days*,* my knees start hurting and my legs hurt… a while ago my hair started falling” P14*,* 26*
Establishing dietary and physical activity routines	Psychological capability	Behavioral regulation	*“I eat six small meals a day*,* the meal itself is divided…I eat protein first*,* fiber*,* then carbs*,* and I don’t eat sweets” P7*,* 44*
			*“I focus on proteins and small meals*,* I stopped sweets*,* fried food*,* ready-made food. Everything changed toward healthier home food” P12*,* 50*
			*“Every day I walk at least 7*,*000 steps. Even if I am busy*,* I leave everything and go out to walk*,* I also do exercises and dancing at home sometimes” P10*,* 32*
			*“Every two or three days I walk to my mother’s house about 25-minute walk*,* I also do exercises at home using YouTube videos” P15*,* 37*
			***“****I would be lying if I said I was committed to exercise*,* one day I do it*,* one day I don’t… about once a week I do cardio and body-toning exercises at home through videos” P19*,* 23*
Limited postoperative follow-up and healthcare access	Physical opportunity	Environmental context and resources	*“I see the nutritionist regularly*,* but the program she gave me was vague*,* I wanted someone to tell me exactly what to eat day by day…the consultation wasn’t enough*,* I wanted direct access for questions” P6*,* 46*
			*“I follow up with the dietitian*,* but I haven’t gone for a month or two because my appointment was cancelled and I forgot to reschedule” P9*,* 22*
			*“My sister and I who also had the surgery had questions about the food that was not suitable for us*,* we tried calling the hospital and nobody answered*,* we did not know who to ask … and the appointments to my doctor are very far apart… I wish they would improve the guidelines and contact” P19*,* 23*
Time, resource, and infrastructure constraints to physical activity	Physical opportunity	Environmental context and resources	*“I have grandchildren*,* I also have to take care of my mother-in-law*,* I have limited time*,* I can’t go to the gym” P7*,* 44*
			*“I was signed up at a gym*,* but it was a bit far from me*,* so I preferred to continue exercising at home” P13*,* 36*
			*“I don’t have time to exercise …work*,* coming back to take care of home*,* cooking and so on*,* I don’t have time” P18*,* 32*
			*“The financial situation is not the best for me to go to a gym… I have a problem with the gym; it’s far for me… we live in a village you know there are no walking paths around” P19*,* 23*
Food environment and social eating context	Physical opportunity	Environmental context and resources	*“At first I wouldn’t go to gatherings because I couldn’t eat and it felt awkward” P1*,* 24*
			*“When I’m at family gatherings*,* I eat what suits me*,* they keep trying to make me eat and I tell them I can’t” P3*,* 46*
			*“The sweets are very tempting these days during Ramadan… in our gatherings there are always sweets and snack foods” P15*,* 37*
Social support as enabler of behavior-change	Social opportunity	Social influences	*“Everyone stood by me; my husband*,* my family*,* my in-laws*,* with weight-loss surgery*,* you need people to support you” P1*,* 24*
			*“I was lucky because I had people around me*,* a doctor*,* a physiotherapist*,* and family members who supported me. Close professional follow-up after surgery is essential; after the surgery*,* there should be monthly and close follow-up. When the nutritionist is available and follows the patient closely*,* it makes a huge difference” P7*,* 44*
			*“In our family many people did the surgery. We all look at each other*,* we see what happens with each other*,* each one benefits from the other” P14*,* 26*
			*“Everyone supported me*,* thank God. My husband tells me*,* ‘Get up and move*,* that’s better for you*,* take your vitamins… my mother supports me at gatherings and tells people not to make me eat sweets” P15*,* 37*
			*“What really helped me was my nutritionist’s close follow-up… even on WhatsApp*,* whenever I message her*,* she replies right away” P17*,* 32*
Social norms, pressure, and comparison	Social opportunity	Social influences	*“My family? No one follows a healthy lifestyle*,* if they did*,* I would have lost weight a long time ago” P4*,* 50*
			*“People told me I should have lost more weight by now. My friends keep saying people who had the surgery lose more weight” P15*,* 37*
			*“They used to pressure me to eat*,* I used to feel embarrassed*,* after surgery I set boundaries*,* I learned how to say no; that’s all I can eat” P7*,* 44*
			*“In the first month I only lost about 6 kg. Others said they lost 10 or 15 kg. My three cousins and I did the surgery together. It shows on them more than on me. I did lose a lot*,* but not the way I wanted” P14*,* 26*
Health-related consequences and concerns of relapse	Reflective motivation	Beliefs about consequences	*“I was constantly tired*,* I had sleep apnea and couldn’t breathe*,* I couldn’t take care of my house or my baby*,* the doctor told me I needed to lose weight” P2*,* 28*
			*“I don’t want to go back to how I was before. As long I see that my health is good*,* why should I ruin it? " P3*,* 46*
			*“My biggest fear is that I will start eating again and regain weight*,* so I keep myself controlled” P6*,* 46*
			*“I have to commit*,* so I wouldn’t ruin my results*,* I was waiting to get to this point and for my health to get better” P7*,* 44*
Perceived self-efficacy and behavioral control	Reflective motivation	Beliefs about capabilities	*“My self-support and encouragement are crucial to stick to the guidelines and maintain a healthy lifestyle” P3*,* 46*
			*“I know my limits; I know what suits me and what doesn’t… I follow the instructions in a way that works for me” P10*,* 32*
			*“I can control it… even at gatherings*,* I can stop*,* I know my limits” P13*,* 36*
Health goals and behavioral intentions	Reflective motivation	Goals/Intentions	*“I want to go back to exercising and stay committed to my health” P3*,* 46*
			*“I would like to exercise and reach my Ideal weight” P6*,* 46*
			*“I want to live a healthy life. Healthy eating and movement became my lifestyle” P13*,* 36*
			*“I want to regulate my sleeping hours… also continue losing weight” P14*,* 26*
Visible progress and external feedback	Automatic motivation	Reinforcement	*“Seeing my weight-loss and improved test results motivates me to keep going” P4*,* 50*
			*“I look younger now. Before I looked much older than my age*,* now I dress my age*,* I wear size 40*,* this motivates me to continue” P11*,* 34*
			*“I wasn’t expecting to lose this much weight*,* I lost around 35 kilos*,* everyone tells me I look much younger” P17*,* 32*
Emotional distress and adjustment following surgery	Automatic motivation	Emotion	*“At the beginning*,* it was hard. I felt depressed because I cook everything with my hands and couldn’t eat like the rest of the family. But it was only at the start*,* and it passed” P12*,* 50*
			*“Before*,* food was happiness for me. Now you feel like something is missing. There is something different” P14*,* 26*
			*“The first month was tiring for me*,* I felt exhausted*,* depressed*,* and I didn’t want to see anyone… but it passed” P18*,* 32*
Psychological well-being, self-confidence, and satisfaction	Automatic motivation	Emotion	*“My self-confidence increased*,* I always loved my body even when I was overweight and now*,* I am more confident” P2*,* 28*
			*“I’m happy with myself*,* I feel like a new person… I feel more carefree*,* overall*,* I feel fine and alive” P7*,* 44*
			*“I’m very happy. If I could go back in time*,* I would do it again… my mood definitely changed for the better*,* I’ve come to love life more” P11*,* 34*
Abbreviations: COM-B = Capability, Opportunity, Motivation–Behavior; TDF = Theoretical Domains Framework. ^&^Participant quotations are identified by participant number (P) and age in years (e.g., P10, 32 indicates participant 10, aged 32 years).			

### Capability

#### Physical Capability: Physical skills

##### Physical Functioning and Stamina

Nineteen participants described improved physical functioning, including increased mobility, reduced bodily pain, increased energy, improved sleep and greater ability to perform daily activities. Some participants (*n* = 9) described fatigue, discomfort, and reduced energy in the early postoperative period, but these were described as temporary and improved with recovery.

#### Psychological Capability: Knowledge

##### Knowledge of dietary recommendations

Dietary guidance and nutritional knowledge were described by fifteen participants. Participants demonstrated understanding of key principles such as prioritizing protein intake, consuming small and frequent meals, chewing thoroughly, and separating food from fluid intake. Guidance was generally perceived as clear and practical regarding these recommendations, although some participants described variability in the clarity and level of detail of the guidance they received.

##### Variability in physical activity guidance

Physical activity knowledge was discussed by all participants. Most reported receiving some instruction from surgeons, dietitians and physiotherapists; however, this varied from general advice to more structured recommendations. Some described clear guidance regarding activity progression, such as gradual return to exercise and restrictions on heavy lifting, whereas others received minimal or no specific information. Physical activity guidance was perceived as inconsistent and insufficiently structured, leaving some participants uncertain how to initiate exercise.

##### Self-directed learning and information seeking

Sixteen participants described actively seeking information beyond healthcare guidance, particularly regarding diet and physical activity. Digital sources (e.g., videos, social media, search engines) were used. Several participants (*n* = 7) used ChatGPT for real-time, personalized guidance, such as checking food suitability and supporting eating-related decisions and symptom management.

#### Psychological Capability: Memory, Attention, Decision Processes

##### Cognitive barriers to adherence (e.g., vitamin supplementation)

Challenges with consistent adherence were reported by eight participants, particularly in relation to vitamin supplementation. Forgetting to take vitamins was common despite awareness of their importance, and in some cases was associated with symptoms such as fatigue or dizziness.

#### Psychological Capability: Behavioral Regulation

Eating behaviors were actively regulated, with most reporting structured dietary patterns. These included small, frequent meals, prioritizing protein, and avoiding certain foods such as sweets and fried items. Dietary practices were perceived as routine-based. Physical activity varied; most participants (*n* = 14) described consistent routines, such as daily walking or regular gym attendance, while others reported irregular or no activity.

### Opportunity

#### Physical Opportunity: Environmental Context and Resources

##### Limited postoperative follow-up and healthcare access

Eight participants across postoperative stages described gaps in postoperative follow-up and support. Although initial guidance was provided, continuity of care was limited, including delayed or cancelled appointments, changes in providers, and difficulty contacting healthcare professionals. Some participants (*n* = 4) discontinued follow-up due to these barriers, while others expressed a need for continuous, accessible, and personalized support beyond written materials or single consultations. Limited communication and tailored guidance left participants uncertain about managing their care.

##### Time, resource, and infrastructure constraints to physical activity

Eleven participants described contextual constraints that limited engagement in recommended behaviors, particularly physical activity. These included competing responsibilities such as childcare and caregiving, limited time, and lack of access to suitable facilities or safe environments. These constraints reduced opportunities for regular activity, even among those who expressed motivation to engage.

##### Food environment and social eating context

Food environment and social eating contexts were discussed by nineteen participants. Family gatherings and social occasions were commonly centered around food and required behavioral adjustments. Participants described navigating shared meals, temptations, or eating restrictions during gatherings. Some initially avoided gatherings due to discomfort or perceived inability to eat, while many described adapting over time by setting boundaries, limiting portions, or selecting foods aligned with their dietary needs.

#### Social Opportunity: Social Influences

##### Social support as an enabler of behavior-change

Seventeen participants described support from one or more sources, including family members, peers, and healthcare providers. Family support included emotional encouragement and practical assistance, such as adapting household food practices and helping manage daily responsibilities. In some cases, shared experiences of surgery within the family reinforced adherence. Peer support from others who had undergone surgery was described as motivating and reassuring, providing encouragement and accountability. Support from healthcare providers was emphasized particularly when guidance was clear, accessible, and responsive. Ongoing communication and continuity of care supported adherence and decision-making.

##### Social norms, pressure, and comparison

Fifteen participants described social expectations, comments, and comparisons with others as shaping postoperative experiences. Social norms influenced participants’ behaviors and decisions, including eating in social settings, disclosing surgery, and engaging in physical activity. Several participants described pressure from others to eat, criticism regarding surgery or weight loss, and comments that created pressure to justify their choices or progress. A few participants described social influences on physical activity, including limited engagement in healthy behaviors within their families, privacy concerns related to gym attendance, and perceived expectations that women prioritize their family and household responsibilities over exercise. Comparisons with relatives or peers who had undergone surgery influenced perceptions of progress and outcomes, functioning as both a source of motivation and pressure.

### Motivation

#### Reflective Motivation: Beliefs About Consequences

##### Health-related consequences and concerns of relapse

Health-related concerns were a central driver of both the decision to undergo surgery and subsequent behavior change. All participants described substantial preoperative symptom burden, including physical limitations, comorbidities, and reduced quality of life. Following surgery, improvements in health reinforced commitment to recommended behaviors. For eleven participants, particularly those interviewed after the initial four postoperative months, concerns about weight recurrence and returning to their previous state acted as a strong motivator for continued adherence.

#### Reflective Motivation: Beliefs About Capabilities

##### Perceived self-efficacy and behavioral control

Ten participants described a sense of personal control and confidence in managing postoperative behaviors. They expressed persistence and commitment in regulating eating behaviors and adhering to recommendations. This was reflected in statements about knowing personal limits, following a self-developed system, and maintaining commitment to recommended practices.

#### Reflective Motivation: Goals, Intentions

##### Health goals and behavioral intentions

Future-oriented health and lifestyle goals were expressed by seventeen participants, including intentions to reach a desired weight, improve health outcomes, and engage in physical activity. Some also described personal goals such as pregnancy, improving sleep, or enhancing physical appearance. Intentions were often framed as a commitment to maintaining a healthy lifestyle and sustaining behaviors over time.

#### Automatic Motivation: Reinforcement

##### Visible progress and external feedback

Visible progress and positive feedback were described as motivating. Fifteen participants referred to observable changes such as physical appearance, clothing size, and improved health, which strengthened their desire to continue adherence. External feedback, including compliments from others, further reinforced these changes.

#### Automatic Motivation: Emotion

##### Emotional distress and adjustment following surgery

Eleven participants described emotional distress during the postoperative adjustment period within the first weeks after surgery. Feelings of low mood, frustration, irritability, and sometimes regret were often linked to dietary restrictions and a sense of deprivation. Several participants described gradual adjustment over time.

##### Psychological well-being, self-confidence and satisfaction

Positive psychological and emotional experiences were described by nineteen participants. These included perceived increases in self-confidence, satisfaction with postsurgical outcomes, improved mood, and a more positive sense of self. Participants often linked these feelings to observable changes in appearance and a broader sense of life improvement.

## Discussion

This study explored behavioral determinants of postoperative lifestyle adherence among women from the Arab society following MBS. Themes were mapped across COM-B constructs and TDF domains. Findings highlight that sustained adherence extends beyond the initial postoperative period and is embedded within individual, social, and environmental contexts.

Within capability, participants demonstrated key facilitators related to knowledge, skills, and behavioral regulation, particularly for dietary adherence. Structured guidance appeared to help the development of routines and self-monitoring practices. This is consistent with Yousefi et al. ^23^, who recently identified the development of dietary strategies as a key enabler within behavioral regulation. In contrast, physical activity guidance was perceived as less consistent in our sample, and appeared less actionable, with variability across providers. This may explain why physical activity routines were less established than dietary routines and suggest the need for clearer and more standardized physical activity guidance after MBS [23].

Participants’ use of ChatGPT and similar tools reflects the growing demand for real-time, accessible information-seeking, and reflects the need for personalized and highly available guidance beyond the limited routine scheduled follow-ups. This may be particularly relevant during periods of postoperative uncertainty, when patients seek timely answers to practical questions [24]. Rather than replacing professional support, these tools might support psychological capability through knowledge acquisition and day-to-day decision-making [25]. Clinically guided digital interventions could therefore leverage such tools to provide timely, individualized support while ensuring consistency. This is supported by evidence demonstrating that eHealth and web-based interventions can improve lifestyle behaviors and self-efficacy among bariatric patients [25, 26].

Within opportunity, behavior was shaped by environmental context and resources and social influences. Participants described multiple contextual barriers, including caregiving responsibilities, time constraints, limited follow-up, infrastructure barriers, and culturally embedded food practices within family and social settings. Insufficient continuity of care emerged as a structural barrier, suggesting that postoperative adherence may depend not only on initial education but also on ongoing, accessible, and tailored professional support. These findings align with previous research identifying socioeconomic constraints, competing demands, limited follow-up, and challenges related to eating at social events as barriers to postoperative adherence [27–29]. Our findings further suggest that these contextual barriers may be particularly relevant among Arab women, where structural, cultural, and environmental constraints may interact to limit opportunities for physical activity and sustained lifestyle change [30]. Inocian et al. ^11^ similarly identified culture-specific factors within Middle Eastern contexts, including family influences, religion, lifestyle practices, and healthcare as relevant to bariatric care outcomes.

Notably, social support emerged as a key facilitator within social influences. Family members, peers, and healthcare providers supported adherence through encouragement and practical assistance, reinforcing evidence that social support is a key enabler of long-term adherence [27, 28, 31, 32]. Consistent with Inocian et al. ^11^, these findings suggest that postoperative care should address broader family and social contexts and, where appropriate, involve supportive family members to reinforce dietary and physical activity behaviors. Social comparison also shaped engagement, functioning both as a role model and a contrasting reference point, thereby influencing self-evaluation. Together, these findings suggest that postoperative support must address not only individual behaviors but also the broader social and cultural contexts in which they are practiced.

Within motivation, findings reflected interactions across beliefs about consequences, reinforcement, goals, and emotion. Concerns of returning to a previous state appeared to function as a facilitator for continued adherence to new lifestyle habits after the early recovery phase, as participants began to experience visible results and improved health status. Positive feedback further reinforced adherence by making behavioral benefits tangible. These findings are consistent with evidence identifying beliefs about consequences and self-efficacy as key drivers of behavior change [27, 33].

However, the early postoperative phase was characterized by emotional distress and adjustment challenges, often linked to dietary restrictions and a sense of deprivation. These findings suggest that adherence to a healthy lifestyle after MBS might require emotional adaptation alongside behavioral change. While psychological outcomes often improve following MBS, a subset of patients experience ongoing or fluctuating emotional difficulties [34, 35], suggesting the need for structured emotional support during the early postoperative period, including normalization of emotional responses and strategies to support adaptation [32, 36].

Overall, the findings demonstrate that capability, opportunity, and motivation operated as an interacting system. Improvements in physical capability and perceived health benefits reinforced motivation, while knowledge and guidance varied across participants. Capability and motivation did not always translate into sustained behavior when opportunity was constrained by limited follow-up, competing responsibilities, infrastructure, or social influences. Conversely, family and professional support enhanced opportunity and facilitated adherence.

### Implications for clinical practice

These findings provide a foundation for developing a theory-informed and culturally responsive postoperative intervention that addresses the identified barriers and builds on existing facilitators.

The Behavior Change Wheel (BCW) provides a systematic approach for translating the behavioral influences identified through COM-B and the TDF into intervention design [18, 19]. It guides the selection of intervention functions and behavior change techniques (BCTs), which are specific, observable, and replicable components designed to support behavior change [18, 19].

Based on our thematic findings, potential strategies to facilitate postoperative behavior change include: (1) providing stage-specific education and practical guidance on dietary behaviors and gradual physical activity progression, using instruction, demonstration, and graded tasks; (2) strengthening continuity of care through regular check-ins, reminders, accessible communication with the professional team, and clinically guided digital support; (3) using action planning, problem-solving, and modeled examples to help patients manage family gatherings and social pressure; (4) strengthening social support through family involvement where appropriate, peer-support groups, shared patient experiences, and ongoing communication with healthcare professionals; and (5) supporting early emotional adjustment through information about expected emotional experiences, problem-solving, and techniques to reduce negative emotions [27, 28]. These strategies will inform the subsequent STEP-UP intervention-development and co-design phase, during which the intervention functions and BCTs will be further selected and operationalized with patients and multidisciplinary stakeholders.

### Strengths and Limitations

To our knowledge, this is the first study to apply a COM-B and TDF-informed analysis to postoperative lifestyle adherence among women from the Arab society following MBS, providing theory-informed insights to support culturally responsive intervention development [12]. Nevertheless, several limitations should be considered. First, women were recruited from East Jerusalem district, whose socioeconomic conditions and access to healthcare services may differ from other Arab populations, potentially limiting transferability. Second, interviewees who agreed to participate may have been more engaged in postoperative care or more comfortable participating via remote interviews. Individuals who declined, ceased responding, or did not attend may have had different levels of engagement with postoperative care or different adherence experiences; their perspectives may therefore be underrepresented, introducing potential selection bias. Finally, the women-only sample may also limit transferability to Arab men undergoing MBS, whose postoperative experiences and behavioral determinants may differ, although this focus reflects the particularly high burden of obesity among Arab women in Israel. Future studies should include more diverse Arab communities, socioeconomic backgrounds, and healthcare settings, as well as patients with longer postoperative follow-up.

## Conclusion

Sustained lifestyle adherence following MBS extends beyond early postoperative improvement. It involves ongoing self-regulation within individual, social, healthcare, environmental, and culturally embedded contexts. Among women from the Arab society, these findings underscore the importance of structured, culturally responsive, and theory-informed support within multidisciplinary bariatric care and will inform the next phase on intervention co-design.

## Supplementary Information

Below is the link to the electronic supplementary material.

Supplementary Material 1 (DOCX 19.5 KB)

Supplementary Material 2 (DOCX 21.6 KB)

Supplementary Material 3 (DOCX 22.0 KB)

## Data Availability

The datasets generated and/or analyzed during the current study are not publicly available due to participant privacy and institutional ethical restrictions. Certain study-related materials are available from the corresponding author upon reasonable request and subject to approval by the relevant institutional review board. The study was preregistered in the Open Science Framework (OSF; https://osf.io/6jgkf/overview), and study-related materials are also available on this platform.
