# Supplementary material for: Behavioral Determinants of Lifestyle Modifications Following Metabolic Bariatric Surgery in a Culturally Specific Population: A Qualitative Study
Source: Obes Surg. 2026 Sep 15;36(10):5294–306. doi: 10.1007/s11695-026-08941-6 (PMC13645892; doi:10.1007/s11695-026-08941-6)
Supplement: Supplementary file 1 [file 11695_2026_8941_MOESM1_ESM.docx]

| **Appendix S1.** Mapping of COM-B Constructs to Theoretical Domains Framework and Definitions* | | | |
| --- | --- | --- | --- |
| **COM-B** | **TDF** | **Definition** |  |
| Physical capability | Skills (physical, cognitive and interpersonal skills) | An ability or proficiency acquired through practice |  |
| Psychological capability | Knowledge | An awareness of the existence of something |  |
|  | Memory, attention and decision processes | The ability to retain information, focus selectively on aspects of the environment and choose between two or more alternatives |  |
|  | Behavioral regulation | Anything aimed at managing or changing objectively observed or measured actions |  |
| Physical opportunity | Environmental context and resources | Any circumstance of a person’s situation or environment that discourages or encourages the development of skills and abilities, independence, social competence, and adaptive behavior |  |
| Social opportunity | Social influences | Those interpersonal processes that can cause individuals to change their thoughts, feelings, or behaviors |  |
| Reflective motivation | Professional/social role and identity | A coherent set of behaviors and displayed personal qualities of an individual in a social or work setting |  |
|  | Beliefs about capabilities  Acceptance of the truth, reality, or validity about an ability, talent, or | facility that a person can put to constructive use |  |
|  | Optimism | The confidence that things will happen for the best or that desired goals will be attained |  |
|  | Beliefs about consequences  Acceptance of the truth, reality, or validity about outcomes of a behavior | in a given situation) |  |
|  | Intentions | A conscious decision to perform a behavior or a resolve to act in a certain way |  |
|  | Goals | Mental representations of outcomes or end states that an individual wants to achieve |  |
| Automatic motivation | Reinforcement | Increasing the probability of a response by arranging a dependent relationship, or contingency, between the response and a given stimulus |  |
|  | Emotion | A complex reaction pattern, involving experiential, behavioral, and physiological elements, by which the individual attempts to deal with a personally significant matter or event |  |
| Abbreviations: COM-B = Capability, Opportunity, Motivation–Behavior; TDF = Theoretical Domains Framework.  *Adapted from Michie S, Atkins L, West R. *The Behaviour Change Wheel: A Guide to Designing Interventions*. Silverback Publishing, 2014. | | |  |
