# Supplementary material for: Behavioral Determinants of Lifestyle Modifications Following Metabolic Bariatric Surgery in a Culturally Specific Population: A Qualitative Study
Source: Obes Surg. 2026 Sep 15;36(10):5294–306. doi: 10.1007/s11695-026-08941-6 (PMC13645892; doi:10.1007/s11695-026-08941-6)
Supplement: Supplementary file 2 [file 11695_2026_8941_MOESM2_ESM.docx]

## **Appendix S2: COREQ (COnsolidated criteria for REporting Qualitative research) Checklist**

A checklist of items that should be included in reports of qualitative research. Report the page number in your manuscript where each item is addressed; if an item is not applicable or not reported, note N/A.

| **Topic** | **Item No.** | **Guide Questions/Description** | **Reported on Page No.** |
| --- | --- | --- | --- |
| **Domain 1: Research team and reflexivity** | | | |
| *Personal characteristics* | | | |
| Interviewer/facilitator | 1 | Which author/s conducted the interview or focus group? | 6 |
| Credentials | 2 | What were the researcher’s credentials? e.g. PhD, MD | 6 |
| Occupation | 3 | What was their occupation at the time of the study? | 5-6 |
| Gender | 4 | Was the researcher male or female? | 5-6 |
| Experience and training | 5 | What experience or training did the researcher have? | 5-6 |
| *Relationship with participants* | | | |
| Relationship established | 6 | Was a relationship established prior to study commencement? | 6 |
| Participant knowledge of the interviewer | 7 | What did the participants know about the researcher? e.g. personal goals, reasons for doing the research | 6 |
| Interviewer characteristics | 8 | What characteristics were reported about the interviewer/facilitator? e.g. bias, assumptions, reasons and interests in the research topic | 6 |
| **Domain 2: Study design** | | | |
| *Theoretical framework* | | | |
| Methodological orientation and Theory | 9 | What methodological orientation was stated to underpin the study? e.g. grounded theory, discourse analysis, ethnography, phenomenology, content analysis | 5-7 |
| *Participant selection* | | | |
| Sampling | 10 | How were participants selected? e.g. purposive, convenience, consecutive, snowball | 5 |
| Method of approach | 11 | How were participants approached? e.g. face-to-face, telephone, mail, email | 5 |
| Sample size | 12 | How many participants were in the study? | 5 |
| Non-participation | 13 | How many people refused to participate or dropped out? Reasons? | 5 |
| *Setting* | | | |
| Setting of data collection | 14 | Where was the data collected? e.g. home, clinic, workplace | 6 |
| Presence of non-participants | 15 | Was anyone else present besides the participants and researchers? | 6 |
| Description of sample | 16 | What are the important characteristics of the sample? e.g. demographic data, date | 8, Table 1 |
| *Data collection* | | | |
| Interview guide | 17 | Were questions, prompts, guides provided by the authors? Was it pilot tested? | 5, AppendixS3 |
| Repeat interviews | 18 | Were repeat interviews carried out? If yes, how many? | N/A |
| Audio/visual recording | 19 | Did the research use audio or visual recording to collect the data? | 6 |
| Field notes | 20 | Were field notes made during and/or after the interview or focus group? | 6 |
| Duration | 21 | What was the duration of the interviews or focus group? | 6 |
| Data saturation | 22 | Was data saturation discussed? | 5 |
| Transcripts returned | 23 | Were transcripts returned to participants for comment and/or correction? | N/A |
| **Domain 3: analysis and findings** | | | |
| *Data analysis* | | | |
| Number of data coders | 24 | How many data coders coded the data? | 6-7 |
| Description of the coding tree | 25 | Did authors provide a description of the coding tree? | 6-7 |
| Derivation of themes | 26 | Were themes identified in advance or derived from the data? | 6-7 |
| Software | 27 | What software, if applicable, was used to manage the data? | N/A |
| Participant checking | 28 | Did participants provide feedback on the findings? | N/A |
| *Reporting* | | | |
| Quotations presented | 29 | Were participant quotations presented to illustrate the themes/findings? Was each quotation identified? e.g. participant number | Table 2 |
| Data and findings consistent | 30 | Was there consistency between the data presented and the findings? | 8-13 |
| Clarity of major themes | 31 | Were major themes clearly presented in the findings? | 8-13 |
| Clarity of minor themes | 32 | Is there a description of diverse cases or discussion of minor themes? | 8-13 |

## Developed from: Tong A, Sainsbury P, Craig J. Consolidated criteria for reporting qualitative research (COREQ): a 32-item checklist for interviews and focus groups. International Journal for Quality in Health Care. 2007;19(6):349–57.
