# Supplementary material for: Behavioral Determinants of Lifestyle Modifications Following Metabolic Bariatric Surgery in a Culturally Specific Population: A Qualitative Study
Source: Obes Surg. 2026 Sep 15;36(10):5294–306. doi: 10.1007/s11695-026-08941-6 (PMC13645892; doi:10.1007/s11695-026-08941-6)
Supplement: Supplementary file 3 [file 11695_2026_8941_MOESM3_ESM.docx]

**Appendix S3. Semi-structured interview guide**

A. **Personal information:**

1. Age: _____________
2. Marital status: _______________
3. Place of living: _______
4. Number of children: ___________
5. Education: _____________
6. Areas of occupation: _____________
7. Country of birth: ________________
8. Religion: _________________
9. Type of surgery (e.g., sleeve gastrectomy, one-anastomosis gastric bypass, gastric bypass): _____________
10. Time after surgery (months): __________________
11. Height (cm): ________
12. Current weight (kg): __________
13. Weight prior to surgery (kg): _______________
B. **Opening and general experience**
1. Can you tell me what led you to undergo bariatric surgery?
2. Can you tell me about your experience since having the surgery?

Probe: How do you feel? Can you describe your weight loss? How was the recovery process?

3. What expectations did you have before the surgery? How do you compare these expectations to your lived experience following the surgery?
4. Have you noticed any physical changes since surgery in your overall well-being?

Probe: Have you experienced changes in energy levels, eating limitations, or daily activity?

5. Can you describe any emotional changes you have experienced since surgery, if any?

Probe: Have you experienced changes in mood, stress, motivation, or self-image

C. **Lifestyle behaviors and changes (diet and physical activity)**
1. Could you describe your usual habits related to your physical activity?
2. How would you describe your current eating habits?
3. Have there been any changes in your diet or physical activity routines since the surgery? Probe: If so, how? What prompted the change?
D. **Perceived knowledge and support**
1. What do you know about the recommended diet and physical activity after surgery?
2. Did you receive any guidance about these recommendations?

Probe: If yes, who provided these recommendations, and how?

3. Was the information provided regarding these recommendations sufficient?

Probe: If not, what was missing or could have been improved?

4. Have you looked for any additional information yourself?

Probe: Which sources did you use (e.g., the internet, social media, support groups, or AI tools)?

5. How important is maintaining a healthy lifestyle to you through the recommended eating and physical activity?

Probe: What impact do you believe these behaviors have on your health?

E. **Challenges, barriers, and motivators**
1. What challenges, if any, do you face in following the recommended eating habits (e.g., taking vitamins, portion sizes, meeting protein intake)?
2. What challenges, if any, do you face in following the recommended physical activity (e.g., exercising 30 minutes, 3-5 times a week)?
3. Some people track their food intake, physical activity or weight loss or food intake using apps or smartwatch, do you track any of these?

Probe: If so, how? Do you feel it helps you? if not, why?

4. What helps you stay motivated to follow healthy eating and physical activity recommendations?

Probe: What factors help motivate you (e.g., feeling capable, receiving external support, or seeing results)?

F. **Cultural and environmental factors**
1. What resources, if any, are available to support your physical activity and diet (e.g., access to gyms, safe spaces for walking, healthy food options)?

Probe: How does this affect your ability to follow the recommendations?

2. Do your family, friends, or community members engage in physical activity or follow a healthy lifestyle?

Probe: How does this influence your choices or behavior?

3. In what ways do cultural traditions, family practices, or social expectations influence your eating and physical activity habits?
G. **Planning for the future and closing**
1. Are there any specific lifestyle changes you would like to achieve regarding your health and lifestyle habits?
2. Looking back at your experience, what kind of support, information, or skills would help you better follow physical activity and nutritional guidelines?
3. We are close to finishing the interview. Is there anything else you would like to share or add regarding your experience of maintaining a healthy lifestyle after surgery?
4. Thank you for participating and taking the time to answer the questions.
